# Increased CD271 expression by the NF-kB pathway promotes melanoma cell survival and drives acquired resistance to BRAF inhibitor vemurafenib

**DOI:** 10.1038/celldisc.2015.30

**Published:** 2015-10-27

**Authors:** Abdelali Lehraiki, Michael Cerezo, Florian Rouaud, Patricia Abbe, Marilyne Allegra, Jerome Kluza, Philippe Marchetti, Veronique Imbert, Yann Cheli, Corine Bertolotto, Robert Ballotti, Stéphane Rocchi

**Affiliations:** 1 INSERM, U1065, équipe 1, Centre Méditerranéen de Médecine Moléculaire (C3M), Nice, France; 2 Université de Nice Sophia Antipolis, UFR de Médecine, Nice, France; 3 INSERM, U837, équipe 4 et Faculté de Médecine, Université de Lille II, Lille, France; 4 INSERM, U1065, équipe 4, Centre Méditerranéen de Médecine Moléculaire (C3M), Nice, France; 5 Service de Dermatologie, Hôpital Archet II, CHU, Nice, France

**Keywords:** melanoma, vemurafenib, resistance, CD271, TNFα, NF-κB

## Abstract

Specific BRAFV600E inhibitors (BRAFi) are highly effective in the treatment of melanoma. However, acquired drug resistances invariably develop after the initial response. Therefore, the identification of new mechanisms of acquired resistance gives important clues towards the development of therapies that could elicit long lasting responses. Here we report that CD271 confers resistance to BRAFi in melanoma cells. The expression of CD271 is increased by BRAFi through a stimulation of tumor necrosis factor-alpha (TNFα) secretion that leads to NF-κB signaling pathway activation. CD271 is upregulated in a subset of BRAFi-resistant melanoma cells. The inhibition of TNFα/NF-κB pathway and CD271 silencing restore the BRAFi sensitivity of resistant melanoma cells. Finally, increase of CD271 expression is validated in BRAFi-resistant xenografts tumors and also in tumors from the patients who relapsed under BRAFi. In summary, these results reveal a novel TNFα/NF-κB/CD271 axis whose activation contributes to the acquisition of resistance to BRAFi and therefore may represent a novel therapeutic target to improve the efficacy of therapy in melanoma.

## Introduction

Melanoma is a major deadly form of skin cancer that arises from the malignant transformation of melanocytes. Activative mutations of *BRAF* are the most prevalent genetic alteration in human melanoma, with ≥50% of tumors expressing the BRAFV600E oncoprotein [1, 2]. Recently, the potent and selective BRAFV600E inhibitors (BRAFi), vemurafenib (PLX4032) and dabrafenib (GSK2118436), have shown robust clinical antitumor activity in treating malignant melanoma bearing BRAFV600E mutation [3, 4]. Unfortunately, despite high response rates seen with the BRAFi in BRAFV600E-positive individuals, relapses occur within months following initiation of treatment [5]. Over the past 2 years, tremendous efforts have been directed towards understanding the molecular mechanisms of acquired BRAFi resistances. Relapsing melanomas reactivate pivotal networks, such as the mitogen-activated protein kinase and phosphoinositide 3-kinase pathways [6, 7]. More recently, aberrant expression of splicing isoforms of BRAFV600E [8], or by secondary genetic events, such as overexpression of COT, NRAS mutations or the MEK1C121S mutation [9, 10] has been shown to mediate acquired BRAF inhibitor resistance. However, even among functionally and genetically heterogeneous tumors, common and intrinsic survival mechanisms exist. Later studies suggested that the expression of markers such as ABCB5, JARID1B, CD271 and ABCG2 on specific subpopulation cells are associated with high tumorigenicity and can be responsible for treatment failures and poor clinical outcomes [11, 12]. Given the diversity and complexity of the identified signaling pathways associated with BRAFi resistance, deciphering the implication of these markers in the mechanisms of resistance and in the underlying melanoma progression is still a priority. This is a prerequisite to develop rational strategies aiming at improving the efficacy of treatment regimens and at reducing the risk of melanoma relapses. In this paper, we identified CD271 as a new mechanism of acquired resistance of melanoma cells to BRAFi that involves tumor necrosis factor-alpha (TNFα)/NF-κB pathway activation and sustained CD271 expression.

## Results

### Expression of CD271 in melanoma cell lines and in melanoma cells freshly isolated from patients

**Figure 1:**
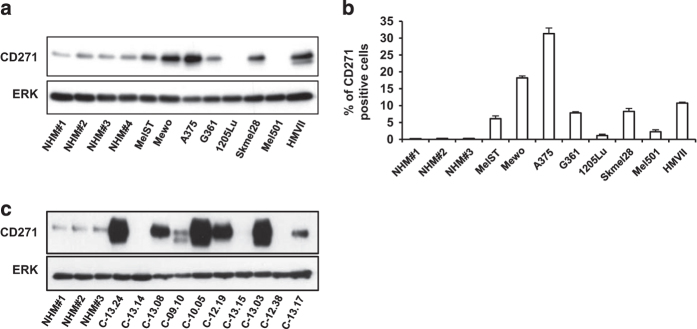
(**a** and **b**) CD271 expression in different melanoma cells and in normal human melanocytes (NHMs) analyzed by western blot and flow cytometry, respectively. (**c**) Western blot expression of CD271 protein in melanoma cells isolated from patients biopsies and in normal human melanocytes.

We have examined CD271 expression in a series of melanoma cells and in normal human melanocytes. The characteristics of melanoma patient cells were indicated on Supplementary Table S1. Western blot (Figure 1a) and flow cytometry (Figure 1b) analyses demonstrated different expression levels of CD271 in melanoma cells. Interestingly, western blot analyses of melanoma cells freshly isolated from patients confirmed results obtained in melanoma cells and showed a strong disparate expression of CD271 in different patient tumor cells (Figure 1c). It should be noted that some melanoma cells isolated from patients expressed very high level of CD271 compared with melanoma cell lines.

### CD271 silencing decreases melanoma cell survival

**Figure 2:**
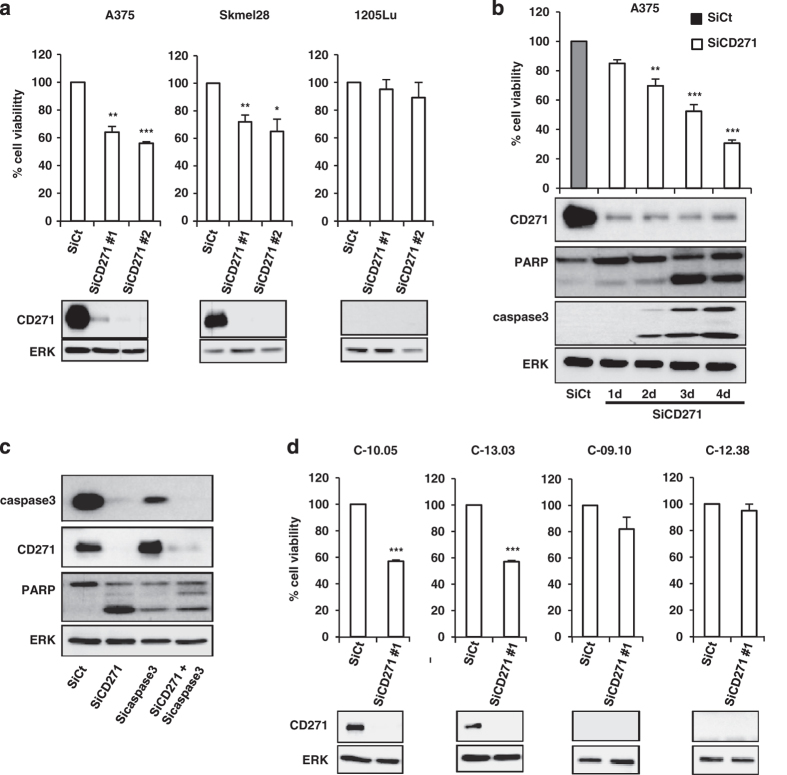
Human melanoma cells (A375, Skmel28 and 1205Lu) (**a** and **b**) or melanoma cells freshly isolated from patients tumors (**d**) were transfected with 50 nm of either siRNA control (SiCt) or siRNA CD271 (SiCD271) for 48 h or at indicated time. At the end of experiment, viable cells were counted using trypan blue dye exclusion method and proteins were extracted and then subjected to western blot using indicated antibodies. ERK serves as a loading control. (**c**) A375 melanoma cells were transfected either with SiCt, SiCD271, Sicasapse-3 or combination of both. Forty-eight hours after transfection, proteins were extracted and CD271, PARP, caspase-3 and ERK were evaluated by western blot. The data showed the mean±s.d. of three independent experiments versus control (*P⩽0.05; **P⩽0.01; ***P⩽0.001).

To investigate the role of CD271 in melanoma cell survival, we silenced CD271 in different melanoma cells. Our results showed that silencing of CD271 by small interfering RNA (siRNA) induced a significant decrease in cell viability of A375 and Skmel28 melanoma cell lines in a time-dependent manner (Figure 2a and b). However, siRNA of CD271 had no effect on cell viability of 1205Lu cells that do not express CD271 (Figure 2a). This decrease in cell viability is mediated by cell death in the form of apoptosis as indicated by PARP and caspase-3 cleavages (Figure 2b). Furthermore, apoptosis induced by CD271 silencing is inhibited when caspase-3 is silenced (Figure 2c). These results have been confirmed by flow cytometry (Supplementary Figure S1A and B). Interestingly, same results were obtained when we silenced CD271 in melanoma cells freshly isolated from patients tumors (Figure 2d). These results confirm the key role of CD271 in the survival of melanoma cells.

### Inhibition of CD271 increases the inhibitory effects of vemurafenib on melanoma cells viability

**Figure 3:**
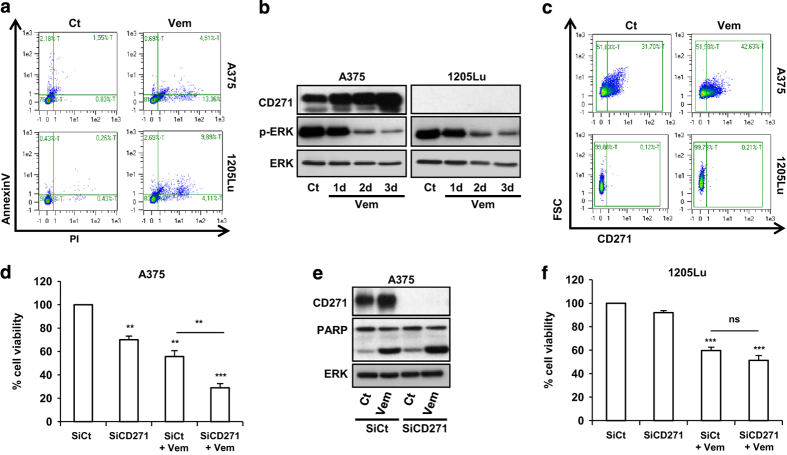
A375 and 1205Lu melanoma cells were treated with vemurafenib 2 μm (Vem) for 48 h or at indicated time. At the end of experiment, cells were co-stained with AnnexinV and PI (propidium iodide) to detect dead cells by flow cytometry (**a**). (**b**) Cell lysates were subjected to western blot for expression level of CD271, phosphorylated ERK (pERK) and total ERK (ERK). (**c**) The percentage of CD271-positive cells was analyzed by flow cytometry. (**d**–**f**) A375 and 1205Lu melanoma cells were transfected either with SiCt or SiCD271. Twenty-four hours after transfection, cells were exposed or not to vemurafenib (Vem) 2 μm as indicated for 24 h. Proteins were extracted and CD271, PARP and ERK were evaluated by western blot (**e**), viable cells were counted using trypan blue dye exclusion method (**d** and **f**). The data showed the mean±s.d. of three independent experiments versus control (**P⩽0.01; ***P⩽0.001).

We investigated the effect of vemurafenib, a specific inhibitor of BRAFV600E on the expression of CD271. As expected, vemurafenib treatment of melanoma cells mutated on BRAFV600E resulted in a significant increase in cell death and a robust inhibition of extracellular signal-regulated kinases (ERK) phosphorylation in all tested cell lines (Figure 3a and b). These effects are concomitant with a significant increase of CD271 expression and in the percentage of CD271 positive in A375 cells that express a basal level of CD271, but not in 1205Lu that displays no basal CD271 (Figure 3b and c). This increase seems to be reversible after drug withdrawal. Indeed, vemurafenib-treated cells revert back to CD271-low expression until reaching a control level after 2 weeks of drug holiday (Supplementary Figure S1C).

Vemurafenib or CD271 silencing decreased viability and increase apoptosis of A375 melanoma cells (Figure 3d and e). Interestingly, the combination of CD271 silencing with vemurafenib further enhanced the apoptotic effects of vemurafenib (Figure 3d and e). However, no effect of CD271 silencing or additive effect with vemurafenib was observed in 1205Lu cell lines that do not express CD271 (Figure 3f). These results suggest that CD271 has a role in melanoma cell survival and that the enrichment of tumor in this population can have a role in tumor-acquired resistance to BRAF inhibitor.

### CD271^+^ cells are resistant to vemurafenib

**Figure 4:**
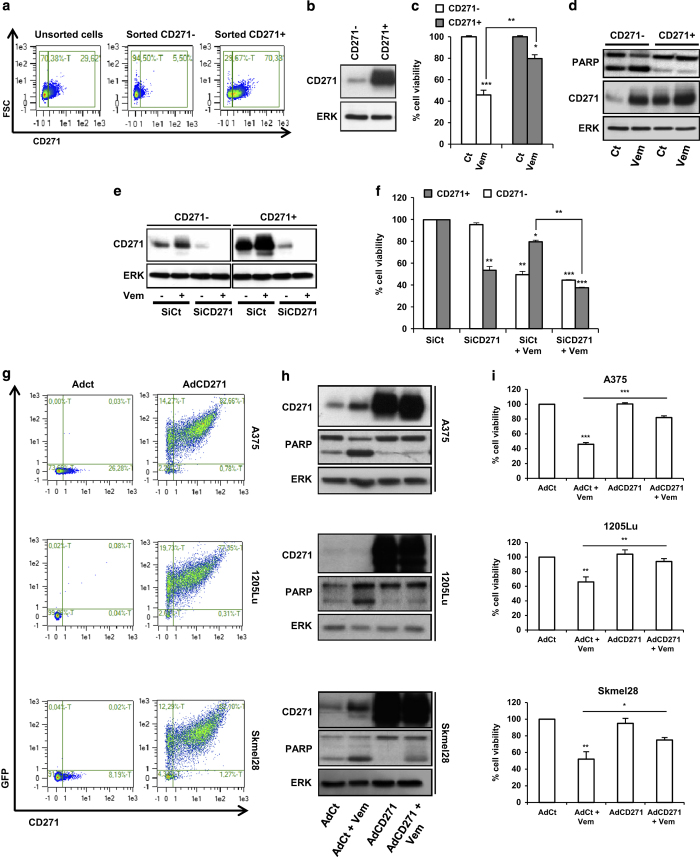
A375 melanoma cells were stained with anti-CD271 antibody and then sorted by flow cytometry. Percentage of CD271-stained cells was determined by flow cytometry (**a**) and CD271 protein level was analyzed by western blot (**b**). After cell sorting, CD271^−^ and CD271^+^ cells were plated and then treated with vehicle (Ct) or vemurafenib (2 μm; Vem) for 48 h. At the end of experiment, viable cells were counted using trypan blue dye exclusion method (**c**) and cell lysates were used for western blotting with indicated antibodies (**d**). CD271^−^ and CD271^+^ cells were transfected either with SiCt or SiCD271. After 24 h of transfection, cells were exposed or not to vemurafenib (2 μm) as indicated for 24 h. Proteins were extracted and CD271 and ERK were evaluated by western blot (**e**) and viable cells were counted using trypan blue dye exclusion method (**f**). A375, 1205Lu and Skmel28 melanoma cells were infected either with adenovirus control (AdCt) or adenovirus CD271-GFP (AdCD271). The infection efficacy was analyzed by flow cytometry (**g**). Twenty-four hours after infection, cells were exposed or not to vemurafenib (2 μm) for 48 h. Proteins were extracted and then subjected to western blot using indicated antibodies (**h**). Viable cells were counted using trypan blue dye exclusion method (**i**). The data showed the mean±s.d. of three independent experiments (*P⩽0.05; **P⩽0.01; ***P⩽0.001) versus control. GFP, green fluorescent protein.

To evaluate the role of CD271 in BRAFi response, we sorted CD271-low population (CD271^−^) and CD271-high population (CD271^+^) by flow cytometry (Figure 4a and b) and analyzed the effect of vemurafenib on these two populations. After cell sorting, we obtained two viable populations (Supplementary Figure S2A). As shown in Figure 4c and d, vemurafenib induced a 60% decrease in CD271^−^ cell viability, associated with an increase of PARP cleavage. However, vemurafenib induced only a 20% in viability of CD271^+^ cells and had no effect on PARP cleavage. This result suggests that CD271^+^ cells are more resistant to vemurafenib treatment than CD271^−^. Importantly, CD271 silencing resensitized CD271+ cells to vemurafenib treatment by inducing death of melanoma cells (Figure 4e and f and Supplementary Figure S2B–D). Again, we observed an increase in CD271^+^ upon exposure of melanoma cells to vemurafenib. In subsequent experiment, using adenovirus construct, we overexpressed CD271 in A375, 1205Lu or Skmel28 cells. The cells overexpressing CD271 became less sensitive to vemurafenib, which confirms the implication of CD271 in the resistance of melanoma cells to BRAFi (Figure 4g–i).

### Increased expression of CD271 has a key role in the acquired resistance to vemurafenib

**Figure 5:**
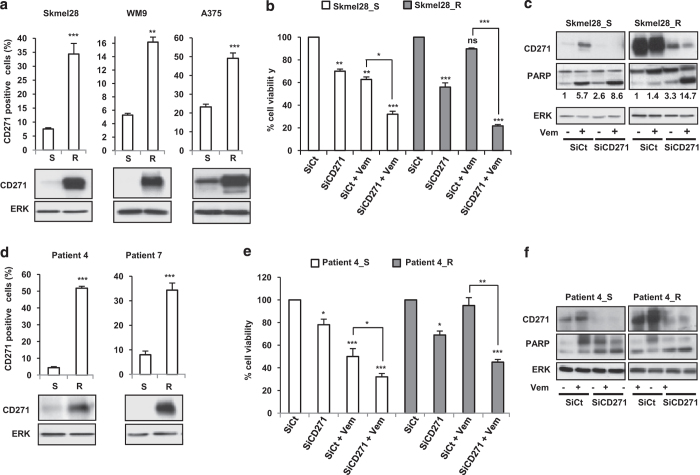
Melanoma cell lines sensitive (S) or resistant (R) to vemurafenib (**a**) or cells isolated from patients biopsies before (S) and after acquired resistance (R) (**d**) were stained with anti-CD271 antibody. The percentage of CD271-positive cells was analyzed by flow cytometry and CD271 protein expression level was determined by western blot. Sensitive (S) or resistant (R) melanoma cells were transfected either with SiCt or SiCD271. Twenty-four hours after transfection, cells were exposed or not to vemurafenib (Vem) 2 μm for 48 h. Viable cells were counted using trypan blue dye exclusion method (**b** and **e**). Proteins were extracted and CD271, PARP and ERK were evaluated by western blot (**c** and **f**). ERK was used as a loading control. The data showed the mean±s.d. versus control of three independent experiments (*P⩽0.05; **P⩽0.01; ***P⩽0.001).

To test the hypothesis of the implication of CD271 in acquisition of resistance to BRAFi therapy, we studied the expression of CD271 in melanoma cell lines rendered resistant. A subset of resistant melanoma cell lines presented a large increase of CD271^+^ cells compared with corresponding parental sensitive cell lines (Figure 5a). Moreover, silencing of CD271 was able to decrease cell viability in basal condition and resensitized resistant cells to the pro-apoptotic effect of vemurafenib (Figure 5b and c). More interestingly, we obtained same results in cells obtained from tumors biopsies before treatment with vemurafenib and after tumors relapse (Figure 5d–f). It is important to note that we tested many tumors biopsies and presented here only those in which CD271 was increased after relapse.

### *In vivo*, CD271 knockdown inhibits tumor progression and abrogates acquisition of resistance

**Figure 6:**
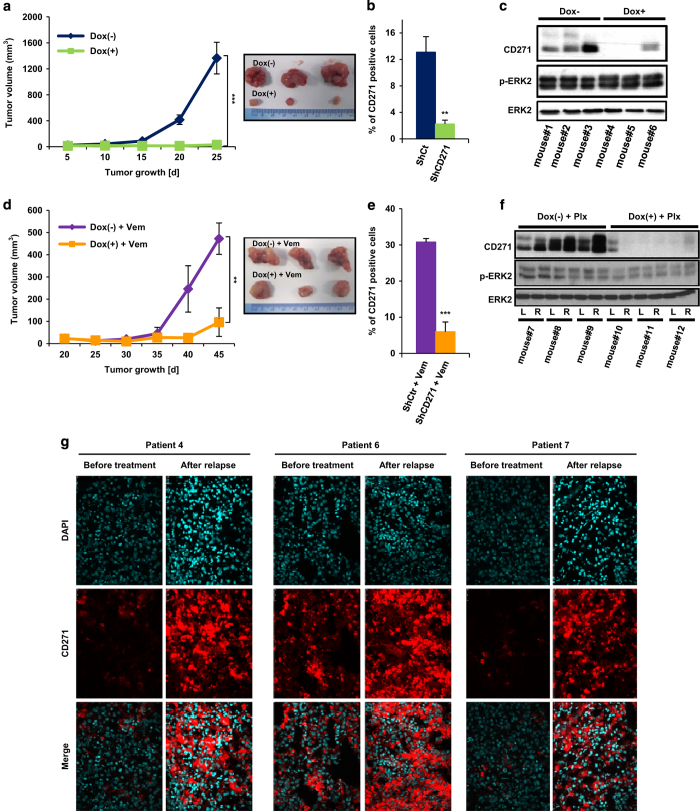
(**a**) A375 cells stably transfected with shRNA CD271 were subcutaneously inoculated in the left dorsal side of nude mice (*n*=6). For induction of shRNA, 1 mg/ml doxycycline was added to the drinking water. Tumor growth was monitored for 25 days and the tumor volume was calculated as described in the Materials and Methods section. At the end of the study, tumors were excised, digested and viable cells obtained by Ficoll gradient centrifugation were then analyzed either by flow cytometry (**b**) or by western blot (**c**). (**d**) A375 cells stably transfected with shRNA CD271 were subcutaneously inoculated in the left dorsal side of nude mice (*n*=6). As previously, shRNA was induced by doxycycline and mice were treated with 45 mg/kg daily of vemurafenib. Tumor growth was monitored for 45 days and the final tumor volume was calculated as described in the Materials and Methods section. At the end of the study, tumors were excised, digested and viable cells obtained by Ficoll gradient centrifugation were then analyzed either by flow cytometry (**e**) or by western blot (**f**). (**g**) Immunofluorescent staining with CD271 (red) and 4',6-diamidino-2-phenylindole (DAPI; blue) in matched pairs of melanoma samples before and after relapse under vemurafenib treatment from three different melanoma patients.

To test the role of CD271 *in vivo*, we established A375 cells expressing doxycycline-inducible small hairpin RNA (shRNA) specific for CD271. After *in vitro* validation of these cells (Supplementary Figure S3), we injected subcutaneously 1×10^6^ of cells transfected with shRNA for CD271 (shCD271) into NUDE mice. One group was provided with doxycycline in water to induce shRNA (Dox+) and the other group with regular water (Dox−). In the vehicle-treated group, tumors progressed steadily until day 25 after injection, whereas in the doxycycline group we observed a marked inhibition of tumors growth (Figure 6a). Flow cytometry (Figure 6b) and western blot analysis (Figure 6c) of the tumors excised at the end of the experiment confirmed effective CD271 knockdown upon doxycycline induction. These data are consistent with the *in vitro* studies and provide evidence that conditional knockdown of CD271 in tumors inhibits their growth *in vivo*.

Our data showed an increase in the number of CD271-positive cells in acquired resistant cells *in vitro* and in relapsed tumors. To investigate the role of CD271 in acquired drug resistance, we generated drug-resistant melanoma tumors by continuous treatment of tumor-bearing mice with vemurafenib. This system mimics the emergence of drug-resistant melanoma in response to drug exposure similar to those seen in patients and has been previously approved for the exploration of drug-resistant mechanism [13]. In our case, we injected subcutaneously 1×10^6^ of cells transfected with shCD271 into nude mice. One group was provided with doxycycline water (Dox+) and the other group with regular water (Dox−). Both groups were continuously treated with 45 mg/kg per day of vemurafenib. Thirty-five days after vemurafenib treatment, drug-resistant tumors emerged in doxycycline-untreated group. Interestingly, we prevented acquired tumors resistance to vemurafenib when CD271 was knockdown upon doxycycline induction (Figure 6d). At the end of experiment, tumors were collected, fragmented and then analyzed for exploration of mechanism of resistance. We assessed the expression of CD271 by flow cytometry and western blot and showed that CD271 is highly represented in resistant tumors (Figure 6e and f).

The relevance of our observations in mice xenografts has been confirmed in patients melanoma biopsies. Indeed, compared with parental tumors before treatment with BRAFi, vemurafenib, a strong increase in the number of cells stained for CD271 had been observed in three melanoma patients that relapsed under vemurafenib (Figure 6g).

### Vemurafenib increases CD271 in melanoma cells by activating NF-κB pathway

**Figure 7:**
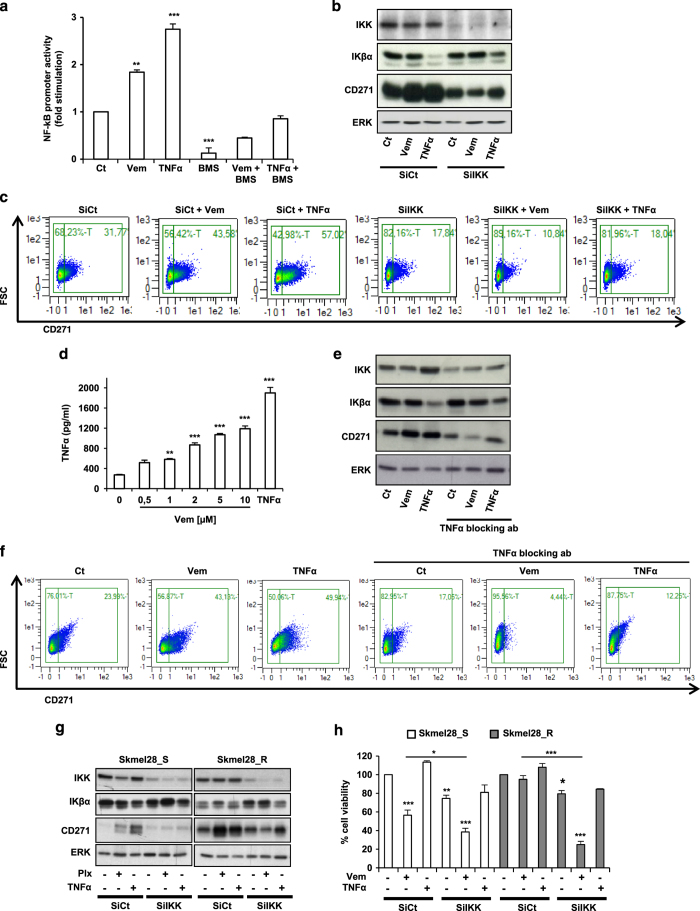
(**a**) A375 melanoma cells were transiently transfected with 0.5 μg of NF-κB promoter and 0.05 μg of pCMVβGal to control the variability in transfection efficiency. Cells were treated for 48 h with vemurafenib 2 μm (Vem), BMS-345541 (1 μm) and TNFα (10 nm) or combinations as indicated. Luciferase activity was normalized to β-galactosidase activity and the results were expressed as fold stimulation of the basal luciferase activity from unstimulated cells. Data are means±s.d. of five experiments performed in triplicate. (**P⩽0.01, ***P⩽0.001) versus control. (**b** and **c**) A375 melanoma cells transfected with 50 nm of either siRNA control (SiCt) or siRNA IKK (SiIKK) were exposed or not to vemurafenib (2 μm) or TNFα (10 nm) for 24 h. Proteins were extracted and CD271, IKK, IKβα and ERK expression were evaluated by western blot. ERK was used as a loading control (**b**). In parallel, the percentage of CD271 was determined by flow cytometry (**c**). (**d**) A375 melanoma cells were treated with vemurafenib at indicated concentration for 48 h and TNFα secretion in medium was detected by enzyme-linked immunosorbent assay. TNFα was used as a positive control. (**e** and **f**) A375 melanoma cells transfected with 50 nm of either siRNA control (SiCt) or siRNA IKK (SiIKK) were exposed or not to vemurafenib (2 μm) or TNFα (10 nm) for 48 h in the presence or the absence of 10 ng of TNFα-blocking antibody. Proteins were extracted and CD271, IKK, IKβα and ERK expression were evaluated by western blot (**e**) and the percentage of CD271 was determined by flow cytometry (**f**). (**g** and **h**) Skmel28-sensitive (Skmel28_S) and -resistant (Skmel28_R) melanoma cells transfected with 50 nm of either siRNA control (SiCt) or siRNA IKK (SiIKK) were exposed or not to vemurafenib (2 μm) or TNFα (10 nm) for 24 h. Proteins were extracted and CD271, IKK, IKβα and ERK expression were evaluated by western blot (**g**) and cell viability was determined using trypan blue dye exclusion method (**h**). The data showed the mean±s.d. of three independent experiments versus control (*P⩽0.05; **P⩽0.01; ***P⩽0.001).

Several studies have shown that NF-κB can have a pivotal role in the maintenance of the malignant state and resistance to therapy [14, 15]. Therefore, we investigated the link between NF-κB, CD271 and vemurafenib. We first examined the effect of this drug on NF-κB promoter activity. We observed that vemurafenib and TNFα (used as a positive control for NF-κB activation) increased NF-κB promoter activity. BMS-345541, a small molecule inhibitor of NF-κB, decreased NF-κB promoter activity in basal condition (Figure 7a) and impaired the activation of NF-κB promoter induced either by vemurafenib or TNFα. These results showed that vemurafenib activated the NF-κB pathway. To evaluate the role of the NF-κB pathway in the CD271 regulation by vemurafenib, we inhibited NF-κB activity using siRNA directed against IKK, a direct activator of NF-κB [16]. As shown by western blot (Figure 7b) and flow cytometry analysis (Figure 7c), both vemurafenib and TNFα were able to increase CD271 expression and the percentage of CD271-positive cells. Interestingly, the silencing of IKK decreased CD271 and abrogated the increase in CD271 induced by either vemurafenib or TNFα. Then, we asked how vemurafenib could activate NF-κB activity and CD271 in melanoma cells. It has been shown that immune cells are able to secrete TNFα, which in turn upregulates CD271 in melanoma cells [17]. TNFα secretion was increased in a dose-dependent manner in response to vemurafenib in A375 cells (Figure 7d). We observed the same increase in TNFα secretion after treatment with other drugs used in melanoma treatment, such as dabrafenib (BRAFi) or trametinib and PD0325901 (MEK inhibitors; Supplementary Figure S4A).

Most importantly, the increase of CD271 induced by both vemurafenib and TNFα was lost in the presence of TNFα-blocking antibody (Figure 7e and f). Taken together, these results demonstrated that vemurafenib controls CD271 in a TNFα/NF-κB-dependent manner in A375 melanoma cells.

We therefore assessed whether NF-κB pathway has a role in resistance to vemurafenib. We first dosed TNFα secreted by sensitive vs resistant melanoma cells and showed that TNFα was increased in resistant cells (Supplementary Figure S4B). We then silenced IKK in sensitive and resistant cells to vemurafenib and treated them with vemurafenib. We observed that both silencing of IKK and treatment with vemurafenib reduced viability of sensitive SKmel28 cells and that combination of IKK silencing and treatment with vemurafenib enhanced the efficacy of vemurafenib on cell viability. More importantly, IKK silencing restores the sensibility to vemurafenib in BRAFi-resistant SKmel28 cells (Figure 7g and h).

Collectively, these results demonstrate the role of theTNFα/NF-κB axis in the regulation of CD271 by vemurafenib and in the acquired resistance to BRAFi.

## Discussion

CD271 is a transmembrane protein that belongs to the tumor necrosis factor receptor superfamily^18^ and is one of the most widely studied melanoma-initiating cell marker studied so far. Several studies including ours demonstrated that CD271 has a crucial role in tumorigenicity maintenance of different cancers including melanoma [19, 20]. Moreover, a CD271/Sox10-positive cell in patient biopsies is associated with poor prognosis for melanoma [21]. However, until today, the role of CD271 in melanoma acquired drug resistance is not completely understood.

In the present study, we demonstrate that CD271 is differently expressed in melanoma cell lines and cells freshly isolated from patients. Treatment of melanoma cells with vemurafenib induced enrichment on CD271^+^ cells. This result suggests that CD271^+^ cells can be highly resistant to targeted therapies. This hypothesis was verified by treatment of highly sorted CD271^+^ cells with vemurafenib. Indeed, our experiment demonstrated that CD271^−^ cells are highly responsive to vemurafenib contrarily to CD271^+^ cells. Interestingly, CD271 knockdown by siRNA induced a marked decrease on cell viability of both melanoma cell line and cells obtained from patients, which means that CD271 has an important role in melanoma cell survival. Of note, CD271 knockdown in 1205Lu melanoma cells that express no detectable level of CD271 do not modify cell viability, whereas CD271 knockdown in SKmel28 and melanoma cells from patient 4 that express a weak but clearly visible expression of CD271 increase the response to vemurafenib. Our findings are in accordance with previous observations regarding the role of CD271 in melanoma [19, 22, 23]. However, these studies did not describe the implication of CD271 in melanoma resistance to targeted therapy. In the present report, we demonstrate that stable knockdown of CD271 using shRNA inhibits tumor xenografts progression and overcomes acquired resistance induced by continuous treatment with vemurafenib. More interestingly, we reported a significant increase in the percentage of CD271^+^ cells in acquired resistant cells *in vitro* and in melanoma patients that relapsed under vemurafenib treatment. All these results identify CD271 as a new mechanism that drives acquired resistance to vemurafenib and provide a potential therapeutic target to overcome acquired resistance in melanoma.

Several studies have shown that NF-κB is constitutively activated in many cancers, and has a pivotal role in the maintenance of the malignant state and resistance to targeted therapy [14, 15]. Furthermore, a recent report identified TNFα/NF-κB pathway as a potent stimulator of CD271 in melanoma cells [17]. We identified NF-κB as a key factor in melanoma acquired resistance due to its ability to sustain the expression of CD271 and cell survival under drug treatment. In this study, we also found that vemurafenib treatment stimulated NF-κB promoter activity and that inhibition of NF-κB pathway led to a marked increase in cell death of vemurafenib-resistant cells and a decrease in the percentage of CD271^+^ cells. Vemurafenib activates NF-κB signaling pathway by stimulating TNFα secretion by treated melanoma cells themselves, leading to autocrine TNFα signaling. Other reports showed that paracrine signaling derived from the microenvironment also has an important role in NF-κB activation in cancer cells and TNFα produced by myeloid cells, particularly macrophages, can promote tumor growth *in vivo* and stimulate tumor cell invasion *in vitro* [14]. All together, these results demonstrate for the first time the pivotal role of CD271 in melanoma acquired resistance and highlights the role of of TNFα/NF-κB signaling pathway in the maintenance of CD271 expression and cell resistance in BRAFi-treated cells.

A recent study demonstrated that melanoma treatment resulted in an enrichment of a therapy-resistant slow-cycling JARID1B cells [13]. Also, we assessed if JARID1B and CD271 subpopulation cells represent the same entity of multidrug-resistant cells. In this report, we identified two different populations expressing either CD271 or JARID1B and only small population of melanoma cells that co-expressed both proteins (Supplementary Figure S5). We obtained same results when we co-stained sensitive and resistant cells with CD271 and ABCB5 (Supplementary Figure S6). These results suggest that melanoma contains an heterogeneous subpopulation, expressing different markers such as CD271, JARID1B or ABCB5, and that are highly resistant to therapies. Moreover, our results demonstrate that these two proteins are regulated by different molecular pathways (Supplementary Figure S5). These observations imply that each tumor is unique and that combination of vemurafinib treatment in patients bearing BRAF mutation with another drug selectively targeting this population seems indispensable to overcome acquired resistance and then tumors relapse. Thus, efforts should be made to develop drugs able to selectively kill these cells.

In summary, our data identified CD271 as a new mechanism of intrinsic drug resistance of melanoma cells. As CD271 has been associated with an increase in stemness markers [20], this observation highlights the pivotal role of stem cell-like population in the resistance to targeted therapy. Obviously, the increase in CD271 expression cannot account for all the cases of acquired BRAFi resistance. Mutations in NRAS, MEK or BRAF amplification also have a pivotal role in the acquisition of the resistance. However, CD271 must be added to the list of membrane receptors that have been involved in BRAFi resistance, such as PDGFR, IGFR or AXL [7, 24, 25].

Furthermore, our results indicate that CD271 expression and BRAFi resistance are supported by TNFα/NF-κB pathway activation, providing a pharmacologically actionable target to overcome drug resistance in melanoma therapy.

## Materials and Methods

### Cell cultures

Normal human melanocyte was obtained from the foreskins of Caucasian children as described previously [26]. All human melanoma cells were grown in Dulbecco’s modified Eagle’s medium (Invitrogen, Carlsbad, CA, USA) supplemented with 7% Fetal calf serum (FCS) and penicillin/streptomycin (100 U ml/50 μg ml^−1^) at 37 °C and 5% CO_2_. Resistant melanoma cells were obtained by chronically treatment with increased concentration of vemurafenib as described previously [27].

### Patient samples

Patient melanoma cells were prepared from biopsies obtained from surgical waste from patients diagnosed for metastatic melanoma at the Nice CHU hospital and treated as reported [28]. Informed consent was obtained from the patients. In brief, biopsy was dissected and digested for 1–2 h with collagenase A (0.33 U ml), dispase (0.85 U ml^−1^) and Dnase I (144 U ml) with rapid shaking at 37 °C. Large debris were removed by filtration through a 70-μm cell strainer. Viable cells were obtained by Ficoll gradient centrifugation and cultured in Roswell Park Memorial Institute medium (Invitrogen) complemented with 7% FCS and penicillin/streptomycin [28].

### Reagents and antibodies

BMS-345541 was purchased from Sigma (Saint-Quentin-Fallavier, France). Vemurafenib, dabrafenib and trametinib were purchased from Euromedex (Souffelweyersheim, France) and TNFα was from PeproTech (Neuilly-Sur-Seine, France). Antibodies against CD271 was purchased from BD Biosciences (Franklin Lakes, NJ, USA), anti-ERK(D-2); anti-cleaved-PARP, anti-cleaved caspase-3, anti-pERK, anti-caspase-3, anti-JARID1B and TNFα blocking antibody were from Cell Signaling Technology Inc. (Beverly, MA, USA). Anti-IKK and anti-IκBα monoclonal antibodies were purchased from Santa Cruz Biotechnology (Santa Cruz, CA, USA). Anti-ABCB5 monoclonal antibody was purchased from Novus Biologicals (Abingdon, UK). Annexin V was purchased from Roche Diagnostic corporation (Indianapolis, IN, USA).

### Western blot assays

Western blots were carried out as described previously [29]. In brief, cell lysates (30 μg) were separated by SDS-polyacrylamide gel electrophoresis, transferred onto a polyvinylidene difluoride membrane, and then exposed to the appropriate antibodies. Horseradish peroxidase-conjugated anti-rabbit, anti-mouse or anti-goat antibodies were from Dakopatts (Les Ulis, France). Proteins were visualized with the ECL system (Amersham, UK). The western blot assays were representative of at least three independent experiments.

### Flow cytometry and fluorescence-activated cell sorting

For surface receptor staining, cells were detached with 2 mm EDTA in phosphate-buffered saline (PBS), centrifuged, incubated for 30 min with primary antibodies in PBS 1% (W/V) bovine serum albumin, washed with PBS/EDTA and then resuspended in 300 μl PBS. Stained cells were used for cell sorting or flow cytometry using FACSAria flow cytometer (BD biosciences, San Jose, CA, USA) or MACSQuant analyzer (Miltenyi biotech, Bergisch Gladbach, Germany), respectively.

### Immunofluorescence

Metastatic melanoma specimens were obtained from the Biobank of the Nice CHU hospital, where they were stored after a diagnosis of melanoma. All patients provided informed written consent. Tissue sections were fixed and permeabilized as described previously [29] before being exposed to anti-CD271 antibody, sections were washed three times with PBS and then incubated for 1 h with 1:1 000 dilution anti-mouse Alexa Fluor 647-labeled secondary antibody (Invitrogen) and mounted using Gel/Mount containing 4',6-diamidino-2-phenylindole (Biomeda Corp, Foster City, CA, USA). Immunofluorescences were examined and photographed with a Zeiss Axiophot microscope (Carl Zeiss SAS, Marly le Roi, France) equipped with epifluorescence illumination.

### Enzyme-linked immunosorbent assay

Cultured medium was collected at different treated conditions and analyzed using a TNFα ELISA kit from PeproTech according to the manufacturer's instructions.

### Transient transfection of small interfering RNA

As described previously [30], a single pulse of 50 nmol/l of siRNA was administrated to the cells at 50% confluency by transfection with 5 μl Lipofectamine RNAiMAX in Opti-MEM medium (Invitrogen) for the indicated time in the figure legends. Control scrambled (SiCt), CD271 and IKK-specific siRNA were purchased from Dharmacon (Velizy-Villacoublay, France).

### Overexpression of CD271

For overexpression of CD271, melanoma cells were stably transfected with a plasmid expressing green fluorescent protein-tagged human CD271 obtained from Vectorbiolabs (Malvern, PA, USA) and selected with puromycin according to the manufacturer's instructions.

### Luciferase reporter assays

A375 melanoma cells were seeded in 24-well dishes, and transient transfections were performed the following day as described previously using the Lipofectamine reagent (Invitrogen) [26]. In brief, cells were transiently transfected with 0.5 μg of NF-κB promoter and 0.05 μg of pCMVβGal to control the variability in transfection efficiency. The transfection medium was changed 6 h later with fresh medium supplemented with 7% FBS and cells were exposed to drugs. At 48 h after transfection, soluble extracts were collected in 50 μl of lysis buffer and assayed for luciferase and β-galactosidase activities. All transfections were repeated at least five times with different plasmid preparations.

### *In vivo* experiments

Animal experiments were carried out in accordance with the Declaration of Helsinki and were approved by a local ethical committee. Animals were maintained in a temperature-controlled facility (22 °C) on a 12-h light/dark cycle and were given free access to food (standard laboratory chow diet from UAR). A375 cells (1×10^6^) expressing shCD271 were subcutaneously inoculated in the left dorsal side of 6-week-old female athymic nude nu/nu mice (Harlan, Gannat, France). For induction of the shRNA in stable clones, 1 mg/ml doxycycline was added to the drinking water of mice 2 days before injection, and the water with doxycycline was changed twice a week. The tumor size was assessed using calipers, and the volume was calculated according to the formula: tumor volume (in mm^3^)=tumor width×tumor length^2^×0.5. At the end of experiment, mice were killed and tumors excised and then analyzed.

### Statistical analysis

Data presented are mean±s.d. of three independent experiments performed in triplicate. Statistical significance was assessed using the Student’s *t*-test except for *in vivo* experiments in which statistical significance was assessed using two-tailed Wilcoxon rank sum test. A value of *P*⩽0.05 was accepted as statistically significant when comparing experimental and control groups.
