# Supplementary figures and images for: Increased CD271 expression by the NF-kB pathway promotes melanoma cell survival and drives acquired resistance to BRAF inhibitor vemurafenib
Source: Cell Discov. 2015 Oct 27;1:15030–. doi: 10.1038/celldisc.2015.30 (PMC4860830; doi:10.1038/celldisc.2015.30)

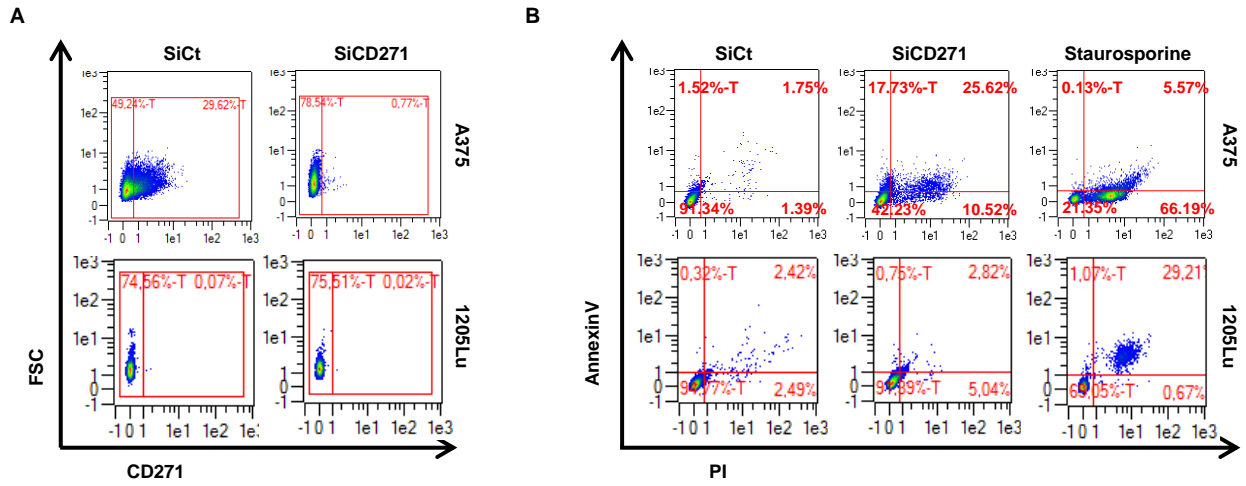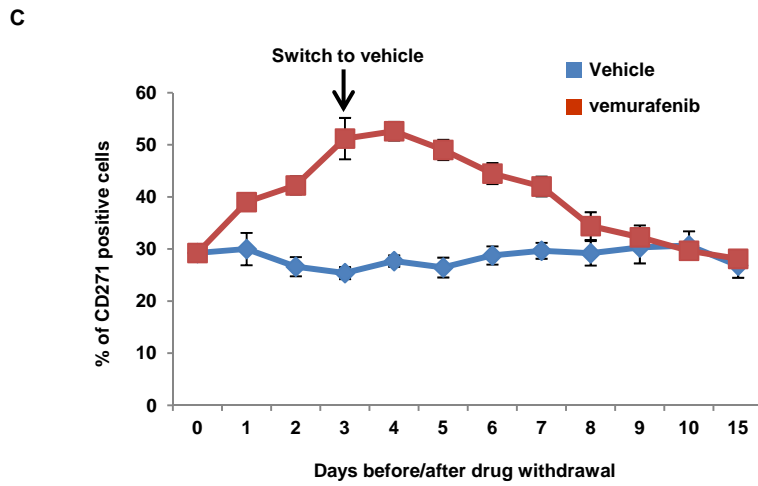

Supplement: Supplementary Figure S1 [file celldisc201530-s1.pdf]

Lehraiki\_Figure 2

A

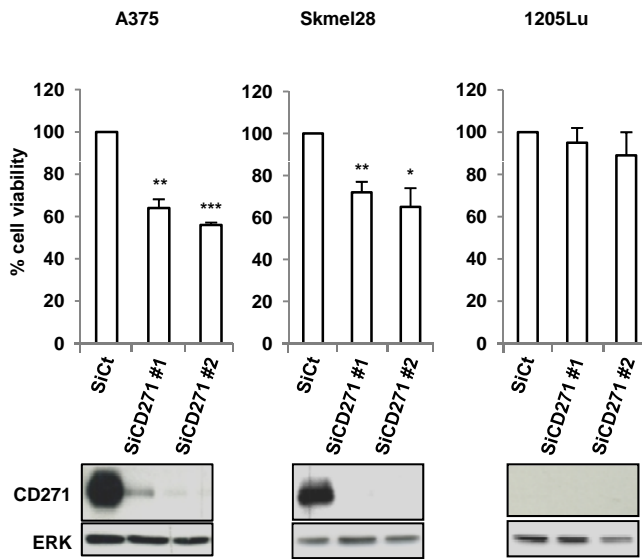

B

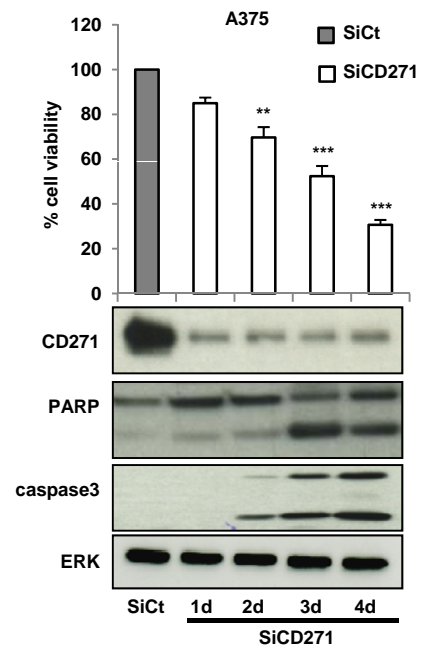

C

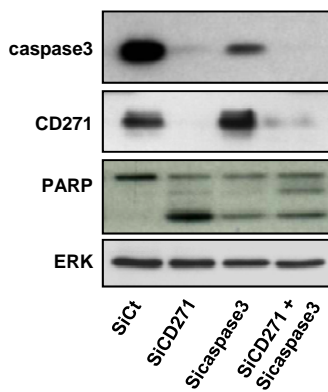

D

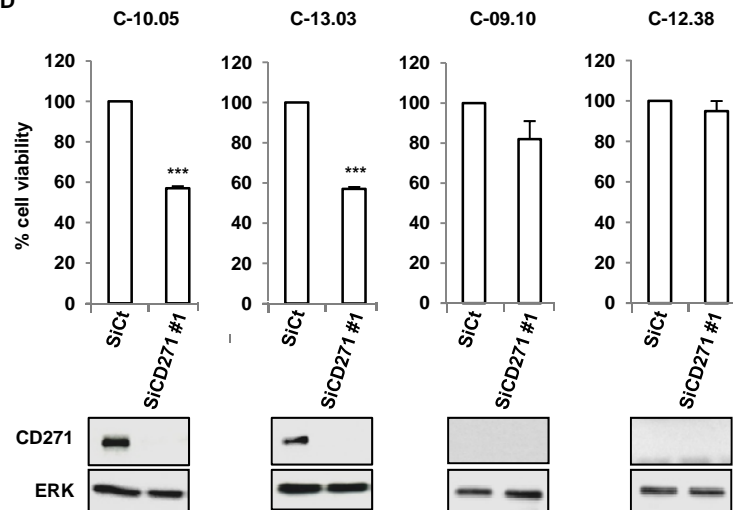

Supplement: Supplementary Figure S2 [file celldisc201530-s2.pdf]

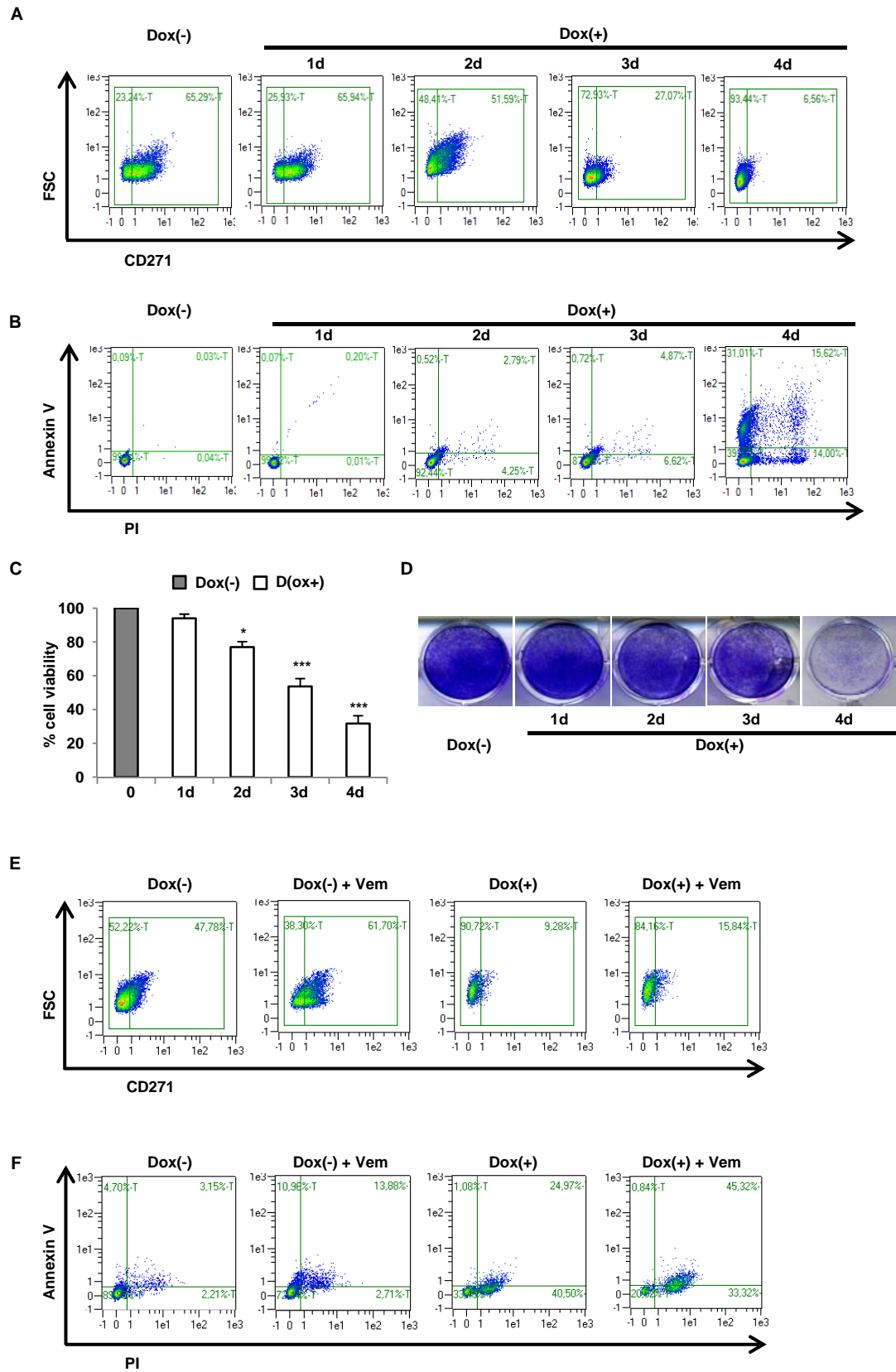

Supplement: Supplementary Figure S3 [file celldisc201530-s3.pdf]

A

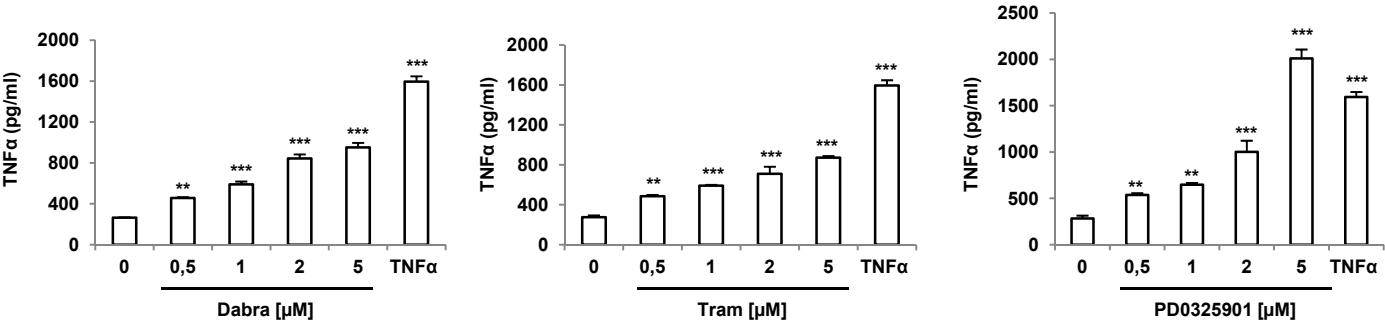

B

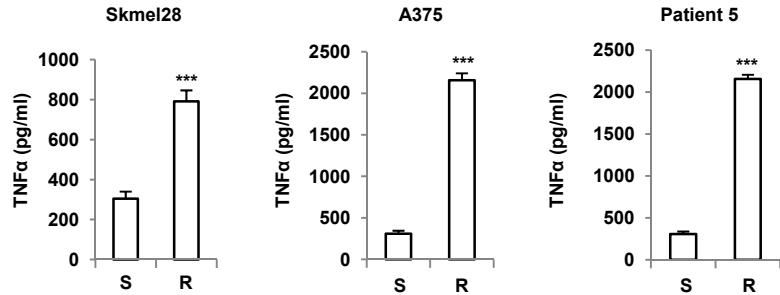

Supplement: Supplementary Figure S4 [file celldisc201530-s4.pdf]

Lehraiki\_Figure 5

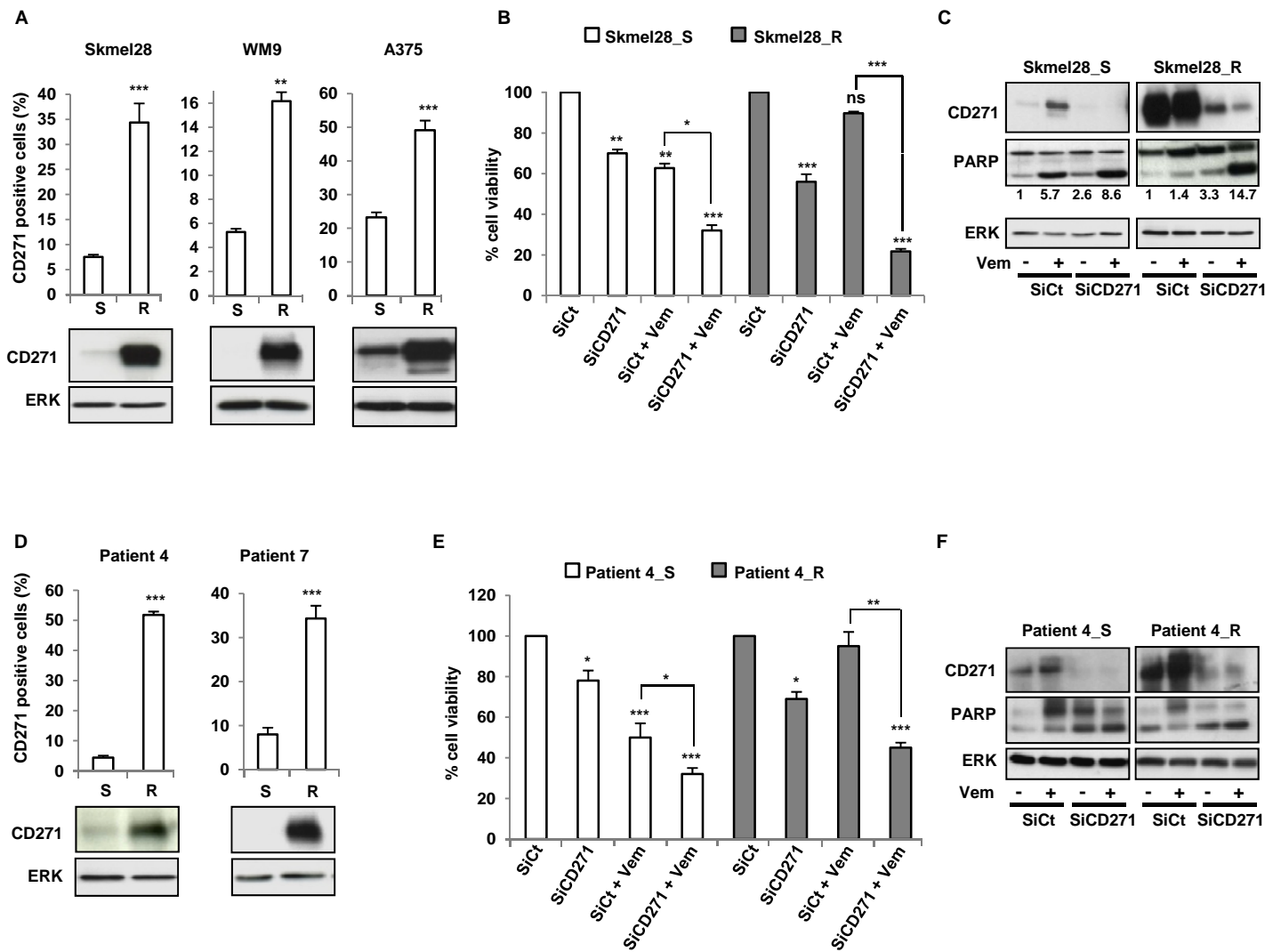

Supplement: Supplementary Figure S5 [file celldisc201530-s5.pdf]

Lehraiki\_Supplementary figure 6

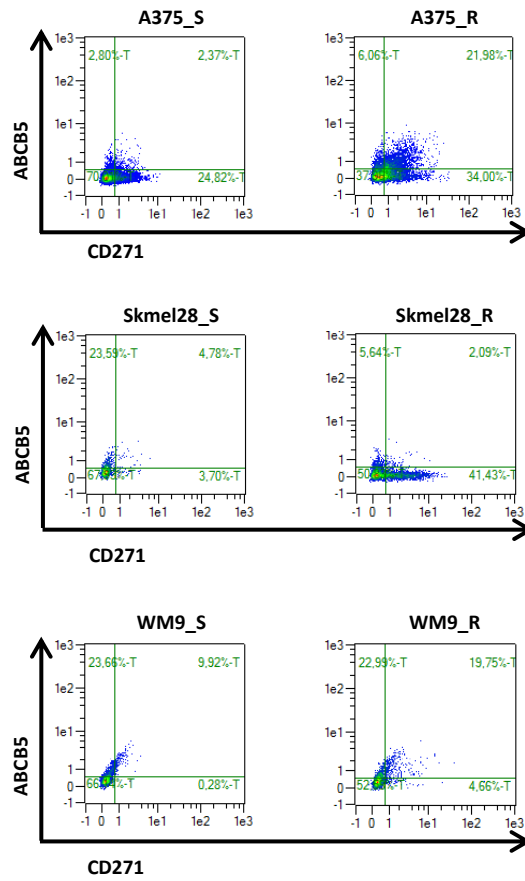

Supplement: Supplementary Figure S6 [file celldisc201530-s6.pdf]
