# Supplementary material for: Increased CD271 expression by the NF-kB pathway promotes melanoma cell survival and drives acquired resistance to BRAF inhibitor vemurafenib
Source: Cell Discov. 2015 Oct 27;1:15030–. doi: 10.1038/celldisc.2015.30 (PMC4860830; doi:10.1038/celldisc.2015.30)
Supplement: Supplementary Table S1 [file celldisc201530-s7.doc]

|  | **cell code** | **mutation** | **sampling** | **melanoma localization** |
| --- | --- | --- | --- | --- |
| **Patient 1** | C-13.24 | BRaf WT / NRas WT | lymphadenectomy | leg |
| **Patient 2** | C-13.14 | BRaf WT / NRas WT | lymphadenectomy | thigh |
| **Patient 3** | C-13.08 | BRaf WT / NRas WT | lymphadenectomy | head |
| **Patient 4** | C-09.10 | BRaf V600E | subcutaneous metastasis | leg |
| **Patient 5** | C-10.05 | BRaf V600E | lymphadenectomy | back |
| **Patient 6** | C-12.19 | BRaf V600E | lymphadenectomy | ND |
| **Patient 7** | C-13.15 | BRaf V600E | lymphadenectomy | ND |
| **Patient 8** | C-13.03 | NRas G13D | lymphadenectomy | ND |
| **Patient 9** | C-12.38 | NRas Q61R | subcutaneous metastasis | back |
| **Patient 10** | C-13.17 | NRas Q61R | lymphadenectomy | leg |

**Lehraiki_Supplementary table1**
