# Supplementary material for: Increased CD271 expression by the NF-kB pathway promotes melanoma cell survival and drives acquired resistance to BRAF inhibitor vemurafenib
Source: Cell Discov. 2015 Oct 27;1:15030–. doi: 10.1038/celldisc.2015.30 (PMC4860830; doi:10.1038/celldisc.2015.30)
Supplement: Supplementary Information [file celldisc201530-s8.doc]

**Supplemental figure legends**

**Supplemental figure 1**

**A, B.** A375, and 1205Lu melanoma cells were transfected with 20µM of either si-RNA control (SiCt) or si-RNA CD271 for 48h. At the end of experiment, cells were either co-stained with AnnexinV and PI (propidium iodide) to detect dead cells by flow cytometry or by an antibody anti-CD271 to evaluate the percentage of CD271 positive cells respectively. Staurosporine was used as positive control for dead cells. **C**. A375 melanoma cells were treated with vemurafenib and then the medium was changed at day 3. We followed the percentage of CD271 expressing cells by flow cytometry before and after drug withdrawal.

**Supplemental figure 2**

**A, B, C.** Sorted CD271- and CD271+ cells were transfected with 20µM of either si-RNA control (SiCt) or si-RNA CD271 for 48h. At the end of experiment, cell lysates were used for Western blotting with indicated antibodies **(A),** viable cells were counted using trypan blue dye exclusion method **(B).** Cells were co-stained with AnnexinV and PI (propidium iodide) to detect dead cells by flow cytometry **(C).** Staurosporine was used as positive control for dead cells. **D.** CD271- and CD271+ sorted cells were stained with AnnexinV and PI (propidium iodide) to ensure that cells are viable after sorting.

**Supplemental figure 3**

A375 cells stably transfected with shRNA CD271 were grown for indicated time in doxycycline-free medium Dox(-) or doxycycline-containing medium (Dox+). At the end of experiment: **A, B.** cells were either co-stained with AnnexinV and PI (propidium iodide) to detect dead cells by flow cytometry (A) or by an antibody anti-CD271 to evaluate the percentage of CD271 positive cells (B) respectively. **C.** viable cells were counted using trypan blue dye exclusion method. **D.** Cells were seeded at the same density in 6-well plates and cultured in the absence or presence of doxycycline at indicated time. The cells were fixed, stained and photographed. **E. F.** Cells were exposed or not to vemurafenib (2µM) for 24h and then co-stained with AnnexinV and PI (propidium iodide) to detect dead cells by flow cytometry (E) or by an antibody anti-CD271 to evaluate the percentage of CD271 positive cells (F) respectively.

**Supplemental figure 4**

**A.** A375 melanoma cells were treated with dabrafenib (Dabra), trametinib (Tram) or PD0325901 at indicated concentration for 48h and TNFα secretion in medium was detected by ELISA. TNFα was used as positive control. **B.** TNFα secretion in medium was evaluated by ELISA in sensitive and resistant melanoma cells (A375 and Skmel28) and in cells from patient.

**Supplemental figure 5**

**A. B.** CD271 and JARID1B expression protein levels in different melanoma cells and in normal human melanocytes were analyzed by Western blot and flow cytometry respectively. **C.** A375 and 1205Lu melanoma cells were treated or not with vemurafenib (2µM) and analyzed for CD271 and JARID1B expression by flow cytometry. **D.** Western blot expression of CD271, JARID1B, p-ERK and ERK proteins from melanoma cells treated different times with vemurafenib (2µM). **E. F.** CD271 and JARID1B were analyzed either by flow cytometry (E) or Western blot (F) in Skmel28 sensitive or resistant cells. **G.** A375 melanoma cells transfected with 20µM or si-RNA control (SiCt) or si-RNA CD271 (SiCD271) were co-stained with anti-CD271 and anti-JARID1B before flow cytometry analysis. **H.** A375 melanoma cells transfected with 20µM of either si-RNA control (SiCt) or si-RNA IKK (SiIKK) were exposed or not to vemurafenib (2µM) or TNFα (10nM) for 48h. Cells were then co-stained with anti-CD271 and anti-JARID1B before flow cytometry analysis. Data shown is representative at least three independently performed experiments.

**Supplemental figure 6**

CD271 and ABCB5 were analyzed by flow cytometry in three sensitive melanoma cells or corresponding resistant cells as described.

**Supplemental table 1: Patient Characteristics.**

Listing of characteristics of melanoma patient cells. Patient, mutation (confirmed by genotyping) and sampling localization are reported. ND: not determined.
